# Supplementary material for: Machine learning identifies T cell receptor repertoire signatures associated with COVID-19 severity
Source: Commun Biol. 2023 Jan 20;6:76. doi: 10.1038/s42003-023-04447-4 (PMC9853487; doi:10.1038/s42003-023-04447-4)
Supplement: Supplementary file 2 — Supplementary Information [file 42003_2023_4447_MOESM2_ESM.pdf]

Figure S1

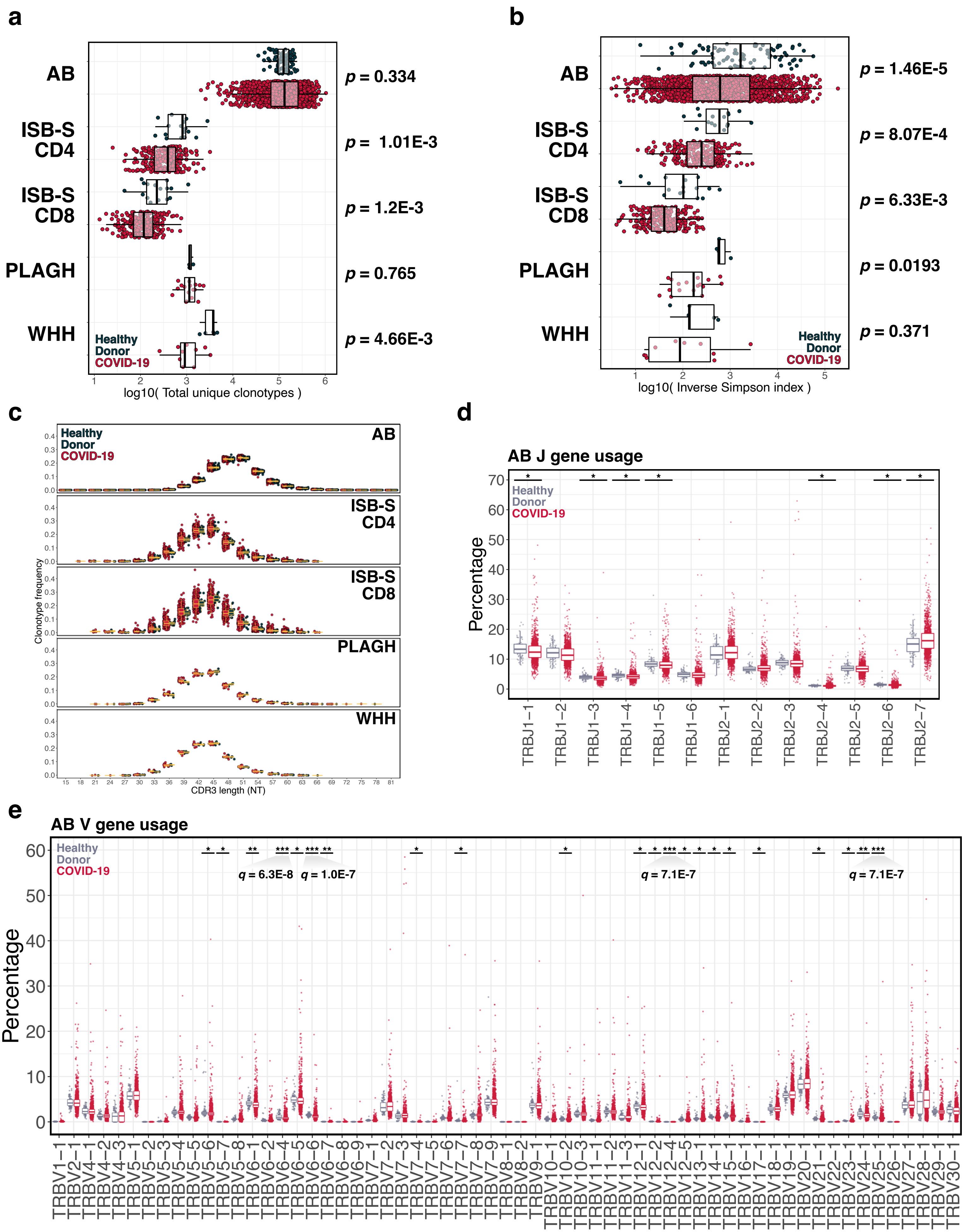

a
AB
ISB-S CD4
ISB-S CD8
PLAGH
WHH
Healthy Donor
COVID-19
p = 0.334
p = 1.01E-3
p = 1.2E-3
p = 0.765
p = 4.66E-3
log10(Total unique clonotypes)
b
p = 1.46E-5
p = 8.07E-4
p = 6.33E-3
p = 0.0193
p = 0.371
log10(Inverse Simpson index)
c
Clonotype frequency
CDR3 length (NT)
d
AB J gene usage
Percentage
TRBJ1−1 TRBJ1−2 TRBJ1−3 TRBJ1−4 TRBJ1−5 TRBJ1−6 TRBJ2−1 TRBJ2−2 TRBJ2−3 TRBJ2−4 TRBJ2−5 TRBJ2−6 TRBJ2−7
e
AB V gene usage
q = 6.3E-8
q = 1.0E-7
q = 7.1E-7
q = 7.1E-7
TRBV1−1 TRBV2−1 TRBV4−1 TRBV4−2 TRBV4−3 TRBV5−1 TRBV5−2 TRBV5−3 TRBV5−4 TRBV5−5 TRBV5−6 TRBV5−7 TRBV5−8 TRBV6−1 TRBV6−2 TRBV6−4 TRBV6−5 TRBV6−6 TRBV6−7 TRBV6−8 TRBV6−9 TRBV7−1 TRBV7−2 TRBV7−3 TRBV7−4 TRBV7−5 TRBV7−6 TRBV7−7 TRBV7−8 TRBV7−9 TRBV8−1 TRBV8−2 TRBV9−1 TRBV10−1 TRBV10−2 TRBV10−3 TRBV11−1 TRBV11−2 TRBV11−3 TRBV12−1 TRBV12−2 TRBV12−4 TRBV12−5 TRBV13−1 TRBV14−1 TRBV15−1 TRBV16−1 TRBV17−1 TRBV18−1 TRBV19−1 TRBV20−1 TRBV21−1 TRBV22−1 TRBV23−1 TRBV24−1 TRBV25−1 TRBV26−1 TRBV27−1 TRBV28−1 TRBV29−1 TRBV30−1

Supplemental Figure Legends

Figure S1. Diversity metrics and gene usages for TCR repertoire datasets.

(a) Boxplot of total unique clonotypes for COVID-19 patients and healthy donors for each repertoire dataset. P-values were obtained using the two-sided Wilcoxon rank-sum test.

(b) Boxplot of inverse Simpson indices for COVID-19 patients and healthy donors for each repertoire dataset. P-values were obtained using the two-sided Wilcoxon rank-sum test.

(c) Boxplot of clonotype frequencies by CDR3 length for COVID-19 patients and healthy donors for each repertoire dataset.

(d) Boxplot of J gene usages for samples from the AB dataset. Gray dots represent healthy donor samples; red dots represent COVID-19 samples. Statistical significance determined using the two-sided Wilcoxon rank-sum test and adjusted using the Benjamini & Hochberg method. * adj. $P < 0.05$, ** adj. $P < 1e-4$, *** adj. $P < 1e-6$.

(e) Boxplot of V gene usages for samples from the AB dataset. Gray dots represent healthy donor samples; red dots represent COVID-19 samples. Statistical significance determined using the two-sided Wilcoxon rank-sum test and adjusted using the Benjamini & Hochberg method. * adj. $P < 0.05$, ** adj. $P < 1e-4$, *** adj. $P < 1e-6$.

Figure S2

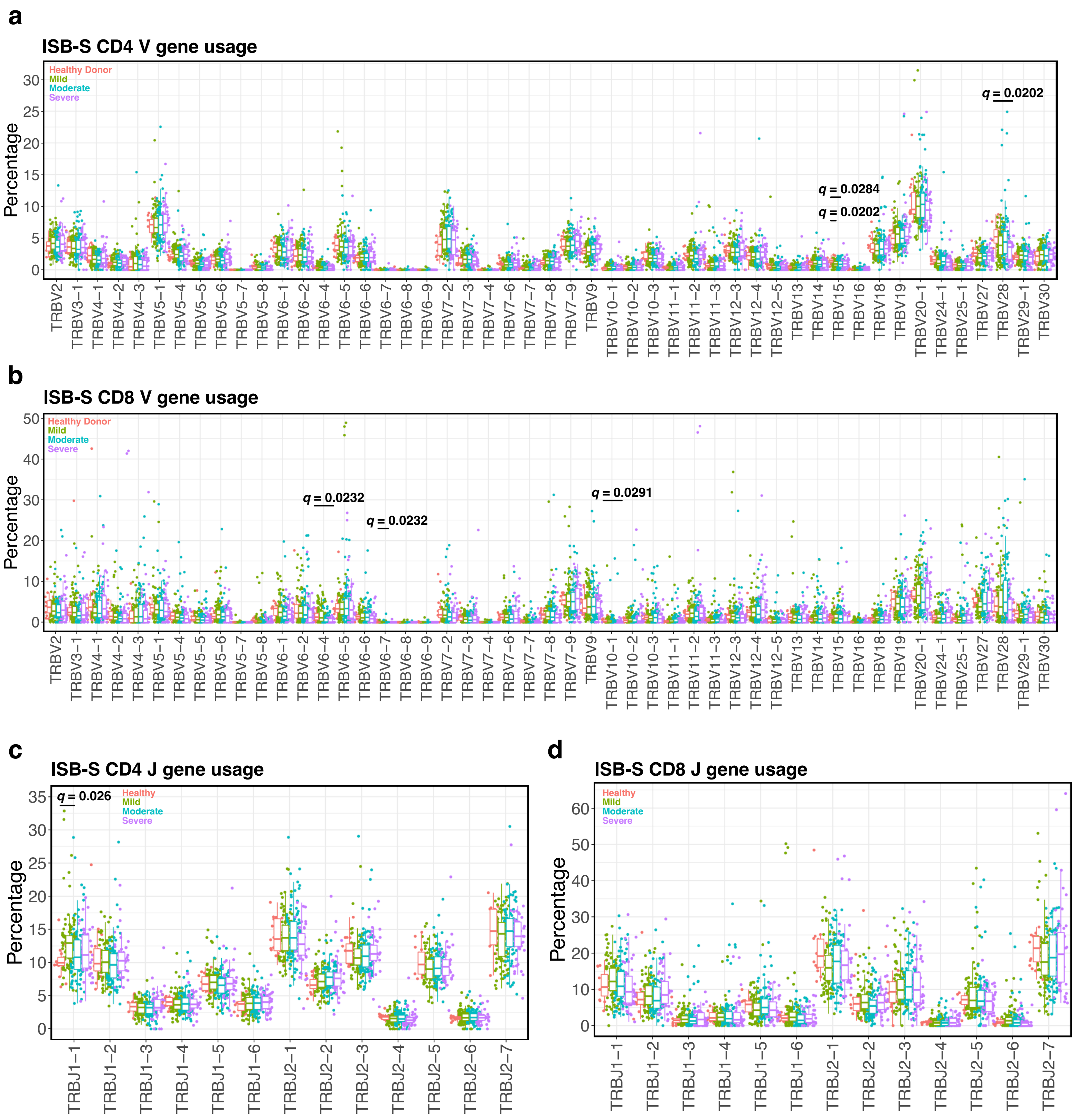
a
ISB-S CD4 V gene usage
Healthy Donor
Mild
Moderate
Severe
Percentage
q = 0.0284
q = 0.0202
q = 0.0202
b
ISB-S CD8 V gene usage
Healthy Donor
Mild
Moderate
Severe
Percentage
q = 0.0232
q = 0.0232
q = 0.0291
c
ISB-S CD4 J gene usage
q = 0.026
Healthy
Mild
Moderate
Severe
Percentage
d
ISB-S CD8 J gene usage
Healthy
Mild
Moderate
Severe
Percentage

Figure S2. V and J gene usages by disease severity for ISB-S datasets.

(a) Boxplot of V gene usages for CD4 samples from the ISB-S dataset. Red dots represent healthy donor samples; green dots represent mild; blue dots represent moderate; purples dots represent severe. Statistical significance determined using the two-sided Wilcoxon rank-sum test and adjusted using the Benjamini & Hochberg method. Adj. $P < 0.05$ labelled on plot.

(b) Boxplot of V gene usages for CD8 samples from the ISB-S dataset.

(c) Boxplot of J gene usages for CD4 samples from the ISB-S dataset.

(d) Boxplot of J gene usages for CD8 samples from the ISB-S dataset.

Figure S3

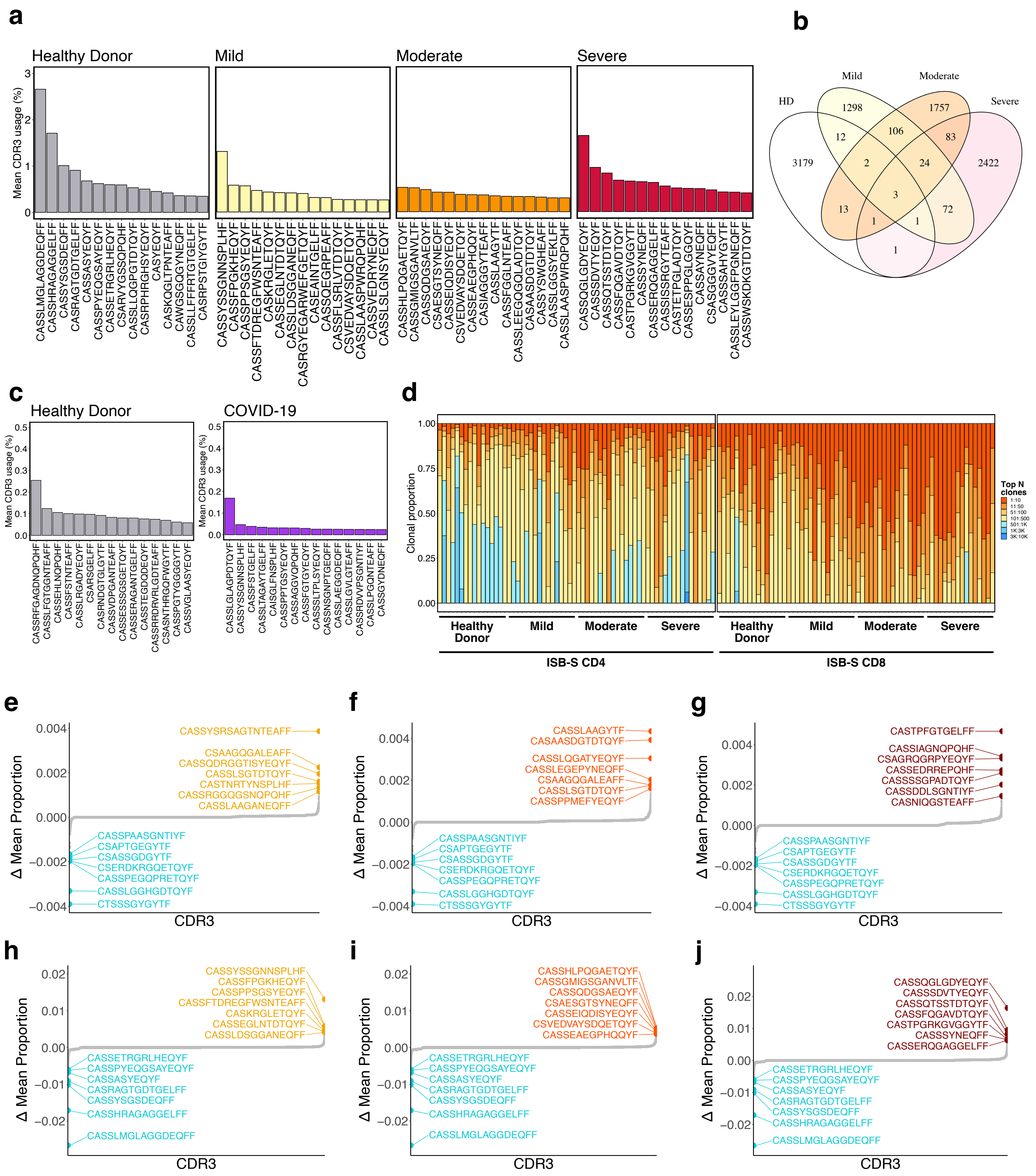

a
Healthy Donor
Mild
Moderate
Severe
Mean CDR3 usage (%)
CASSLMGLAGGDEQFF
CASSHRAGAGGELFF
CASSYSGSDEQFF
CASRAGTGDTGELFF
CASSASYEQYF
CASSPYEQGSAYEQYF
CASSETRGRLHEQYF
CSARVYGSSQPQHF
CASSLQGPGTDTQYF
CASRPHRGHSYEQYF
CASYEQYF
CASKQGLTPNTEAFF
CAWGSGQGYNEQFF
CASSLLFFFRTGTGELFF
CASRPSTGIYGYTF
CASSYSSGNNSPLHF
CASSFPGKHEQYF
CASSPPSGSYEQYF
CASSFTDREGFWSNTEAFF
CASKRGLETQYF
CASSEGLNTDTQYF
CASSLDSGGANEQFF
CASRGYEGARWEFGETQYF
CASEAINTGELFF
CASSQEGRPEAFF
CASSFLSRLVTDTQYF
CSVEDVAYSDQETQYF
CASSLAASPWRQPQHF
CASSVEDRYNEQFF
CASSLSLGNSYEQYF
CASSHLPQGAETQYF
CASSGMIGSGANVLTF
CASSQDGSAEQYF
CSAESGTSYNEQFF
CASSEIQDISYEQYF
CSVEDVAYSDQETQYF
CASSEAEGPHQQYF
CASIAGGGYTEAFF
CASSLAAGYTF
CASSFGGLNTEAFF
CASSLEEGQGQLADTQYF
CASAASDGTDTQYF
CASSYSWGHEAFF
CASSLGGSYEKLFF
CASSLAASPWRQPQHF
CASSQGLGDYEQYF
CASSSDVTYEQYF
CASSQTSSTDTQYF
CASSFQGAVDTQYF
CASTPGRKGVGGYTF
CASSSYNEQFF
CASSERQGAGGELFF
CASISSRGYTEAFF
CASTETPGLADTQYF
CASSESPPGLGGQYF
CASSAYNEQFF
CSASGGGVYEQFF
CASSSAHYGYTF
CASSLEYLGGFPGNEQFF
CASSWSKDKGTDTQYF
b
HD
Mild
Moderate
Severe
3179
1298
1757
2422
12
106
83
2
24
3
13
72
1
1
1
c
Healthy Donor
COVID-19
Mean CDR3 usage (%)
CASSRFGAGDNQPQHF
CASSLFGTGGNTEAFF
CASSEHLNQPQHF
CASSFSTNTEAFF
CASSLRGADYEQYF
CASRSGELFF
CASRNDGTGLGYTF
CASSVDPGANTEAFF
CASSESSSGSGETQYF
CASSERAGANTGELFF
CASSTEGDQDEQYF
CASSRRDRVRLGDTEAFF
CASSNTHRGQFWGYTF
CASSPGTYGGGGYTF
CASSVGLAASYEQYF
CASSLGLAGPDTQYF
CASSYSSGNNSPLHF
CASSFSTGELFF
CASSLTAGAYTGELFF
CAISGLFNSPLHF
CASSPPTGSYEQYF
CASSAGGVQPQHF
CASSFGTGTGYEQYF
CASSSLTPLSYEQYF
CASSNSGNPTGEQFF
CASSLAEGGDEQFF
CASSLGVLGTEAFF
CASSRDVVPSGNTIYF
CASSLPGQNTEAFF
CASSGYDNEQFF
d
Clonal proportion
Top N clones
1:10
11:50
51:100
101:500
501:1K
1K:3K
3K:10K
Healthy Donor
Mild
Moderate
Severe
ISB-S CD4
ISB-S CD8
e
f
g
Δ Mean Proportion
CDR3
CASSYSRSAGTNTEAFF
CSAAGQGALEAFF
CASSQDRGGTISYEQYF
CASSLSGTDTQYF
CASTNRTYNSPLHF
CASSRGGQGSNQPQHF
CASSLAAGANEQFF
CASSPAASGNTIYF
CSAPTGEGYTF
CSASSGDGYTF
CSERDKRGQETQYF
CASSPEGQPRETQYF
CASSLGGHGDTQYF
CTSSSGYGYTF
CASSLAAGYTF
CASAASDGTDTQYF
CASSLQGATYEQYF
CASSLEGEPYNEQFF
CSAAGQGALEAFF
CASSLSGTDTQYF
CASSPPMEFYEQYF
CASTPFGTGELFF
CASSIAGNQPQHF
CSAGRQGRPYEQYF
CASSEDRREPQHF
CASSSSGPADTQYF
CASSDDLSGNTIYF
CASNIQGSTEAFF
h
i
j
CASSYSSGNNSPLHF
CASSFPGKHEQYF
CASSPPSGSYEQYF
CASSFTDREGFWSNTEAFF
CASKRGLETQYF
CASSEGLNTDTQYF
CASSLDSGGANEQFF
CASSETRGRLHEQYF
CASSPYEQGSAYEQYF
CASSASYEQYF
CASRAGTGDTGELFF
CASSYSGSDEQFF
CASSHRAGAGGELFF
CASSLMGLAGGDEQFF
CASSHLPQGAETQYF
CASSGMIGSGANVLTF
CASSQDGSAEQYF
CSAESGTSYNEQFF
CASSEIQDISYEQYF
CSVEDVAYSDQETQYF
CASSEAEGPHQQYF
CASSQGLGDYEQYF
CASSSDVTYEQYF
CASSQTSSTDTQYF
CASSFQGAVDTQYF
CASTPGRKGVGGYTF
CASSSYNEQFF
CASSERQGAGGELFF

Figure S3. Additional CDR3 gene usage statistics.

(a) Bar plots showing the top 15 mean CDR3 usages for patients in the ISB-S CD8 dataset grouped by disease severity (healthy donor = 16, mild = 108, moderate = 93, severe = 49).

(b) Venn diagram showing overlap of top mean CDR3 usages (proportion threshold = 0.0001) for patients in the ISB-S CD8 dataset grouped by disease severity.

(c) Bar plots showing the top 15 mean CDR3 usages for patients in the AB dataset grouped by disease status (healthy donor = 88, COVID-19 = 1,475).

(d) Bar plot depicting relative abundance for groups of top clonotypes by disease condition for sampled repertoires (n = 16 per condition) from ISB-S datasets.

(e) Dotted waterfall plot of CDR3 gene usage differentials between mild disease COVID-19 patients and healthy donors (delta mean proportion) in the ISB-S CD4 dataset. Yellow dots are CDR3 sequences enriched in moderate disease repertoires; light blue dots are CDR3 sequences enriched in healthy donors; grey dots are all other CDR3 sequences.

(f) Dotted waterfall plot of CDR3 gene usage differentials between moderate disease COVID-19 patients and healthy donors in the ISB-S CD4 dataset. Orange dots are CDR3 sequences enriched in moderate disease repertoires.

(g) Dotted waterfall plot of CDR3 gene usage differentials between severe disease COVID-19 patients and healthy donors in the ISB-S CD4 dataset. Red dots are CDR3 sequences enriched in severe disease repertoires.

(h) Dotted waterfall plot of CDR3 gene usage differentials between mild disease COVID-19 patients and healthy donors in the ISB-S CD8 dataset. Yellow dots are CDR3 sequences enriched in mild disease repertoires.

(i) Dotted waterfall plot of CDR3 gene usage differentials between moderate disease COVID-19 patients and healthy donors in the ISB-S CD8 dataset. Orange dots are CDR3 sequences enriched in moderate disease repertoires.

(j) Dotted waterfall plot of CDR3 gene usage differentials between severe disease COVID-19 patients and healthy donors in the ISB-S CD8 dataset. Red dots are CDR3 sequences enriched in severe disease repertoires.

Figure S4

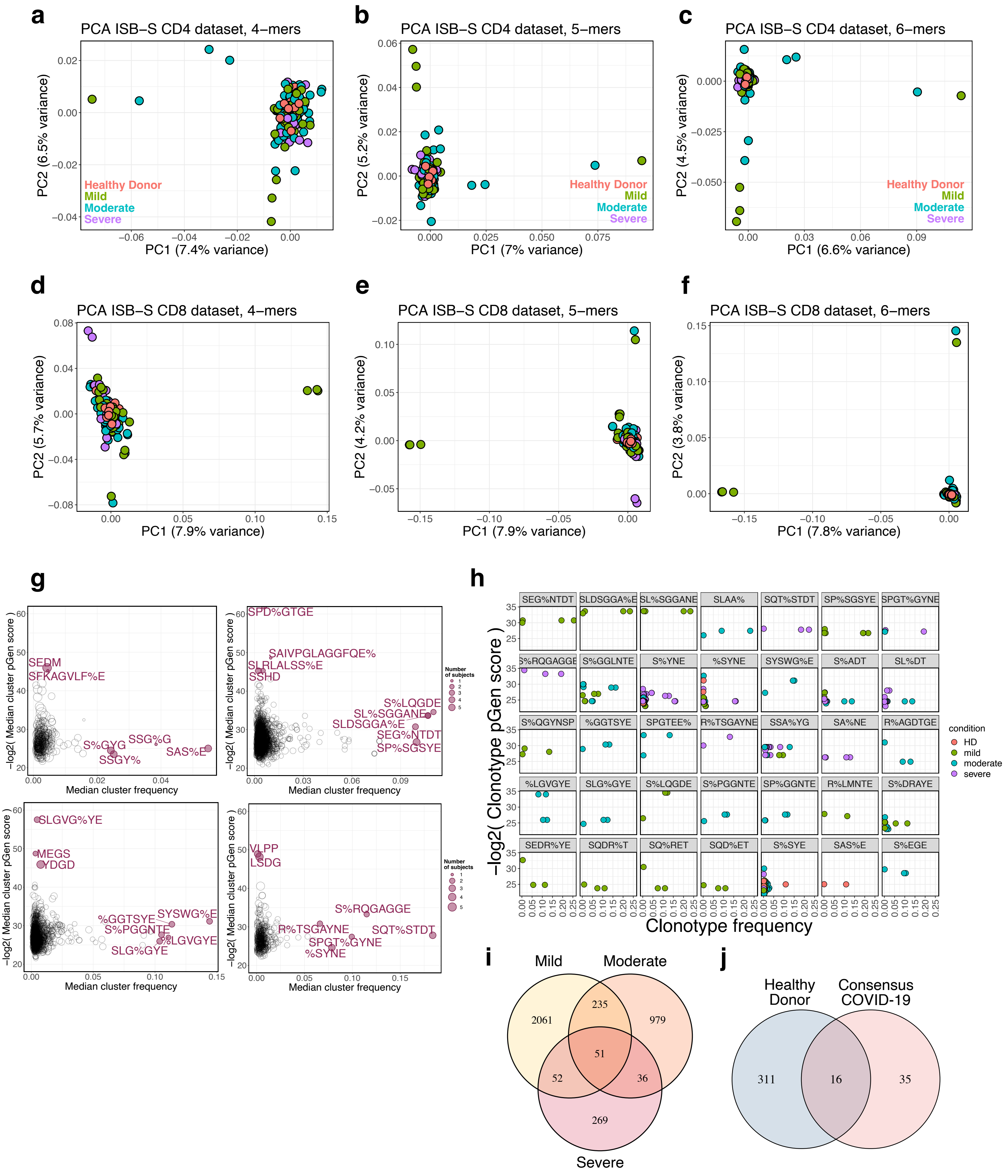
a
PCA ISB−S CD4 dataset, 4−mers
PC2 (6.5% variance)
PC1 (7.4% variance)
Healthy Donor
Mild
Moderate
Severe
b
PCA ISB−S CD4 dataset, 5−mers
PC2 (5.2% variance)
PC1 (7% variance)
Healthy Donor
Mild
Moderate
Severe
c
PCA ISB−S CD4 dataset, 6−mers
PC2 (4.5% variance)
PC1 (6.6% variance)
Healthy Donor
Mild
Moderate
Severe
d
PCA ISB−S CD8 dataset, 4−mers
PC2 (5.7% variance)
PC1 (7.9% variance)
e
PCA ISB−S CD8 dataset, 5−mers
PC2 (4.2% variance)
PC1 (7.9% variance)
f
PCA ISB−S CD8 dataset, 6−mers
PC2 (3.8% variance)
PC1 (7.8% variance)
g
−log2(Median cluster pGen score)
Median cluster frequency
SEDM
SFKAGVLF%E
S%GYG
SSG%G
SSGY%
SAS%E
SPD%GTGE
SAIVPGLAGGFQE%
SLRLALSS%E
SSHD
S%LQGDE
SL%SGGANE
SLDSGGA%E
SEG%NTDT
SP%SGSYE
Number of subjects
SLGVG%YE
MEGS
YDGD
%GGTSYE
SYSWG%E
S%PGGNTE
%LGVGYE
SLG%GYE
VLPP
LSDG
S%RQGAGGE
R%TSGAYNE
SQT%STDT
SPGT%GYNE
%SYNE
h
−log2(Clonotype pGen score)
Clonotype frequency
SEG%NTDT
SLDSGGA%E
SL%SGGANE
SLAA%
SQT%STDT
SP%SGSYE
SPGT%GYNE
S%RQGAGGE
S%GGLNTE
S%YNE
%SYNE
SYSWG%E
S%ADT
SL%DT
S%QGYNSP
%GGTSYE
SPGTEE%
R%TSGAYNE
SSA%YG
SA%NE
R%AGDTGE
%LGVGYE
SLG%GYE
S%LQGDE
S%PGGNTE
SP%GGNTE
R%LMNTE
S%DRAYE
SEDR%YE
SQDR%T
SQ%RET
SQD%ET
S%SYE
SAS%E
S%EGE
condition
HD
mild
moderate
severe
i
Mild
Moderate
Severe
2061
235
979
51
52
36
269
j
Healthy Donor
Consensus COVID-19
311
16
35

Figure S4. Additional k-mer and motif analyses.

(a) PCA of 4-mer representations of TCR repertoires from the ISB-S CD4 dataset.

(b) PCA of 5-mer representations of TCR repertoires from the ISB-S CD4 dataset.

(c) PCA of 6-mer representations of TCR repertoires from the ISB-S CD4 dataset.

(d) PCA of 4-mer representations of TCR repertoires from the ISB-S CD8 dataset.

(e) PCA of 5-mer representations of TCR repertoires from the ISB-S CD8 dataset.

(f) PCA of 6-mer representations of TCR repertoires from the ISB-S CD8 dataset.

(g) Median frequency and pGen scores of COVID-19 and healthy donor associated T cell clusters from GLIPH2 analysis of the ISB-S CD8 dataset, grouped by disease condition.

(h) Detailed view of frequencies and pGen scores of specific clonotypes associated with high frequency T cell clusters from CD8 dataset. Clonotypes are colored by patient disease condition.

(i) Venn diagram showing overlap of COVID-19-associated T cell clusters for patients in the ISB-S CD8 dataset grouped by disease condition.

(j) Venn diagram showing overlap between consensus COVID-19-associated T cell clusters (taken from intersection of disease conditions) and healthy donors for repertoires in the ISB-S CD8 dataset.

Figure S5

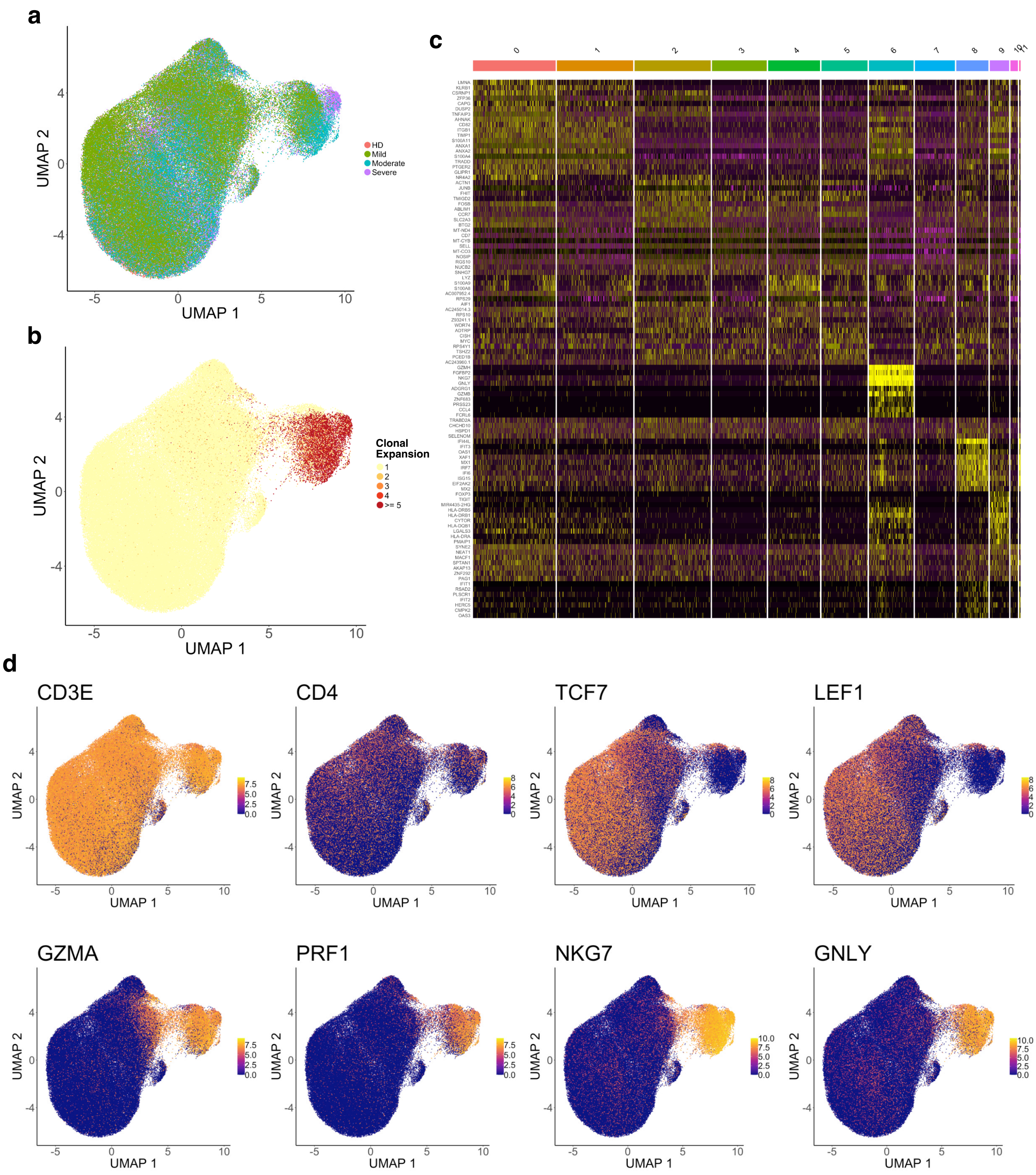
a
b
c
d
UMAP 1
UMAP 2
HD
Mild
Moderate
Severe
Clonal Expansion
1
2
3
4
>= 5
CD3E
CD4
TCF7
LEF1
GZMA
PRF1
NKG7
GNLY

Figure S5. Additional single-cell transcriptional analyses for CD4 T cells.

(a) UMAP visualization of 137,075 CD4 T cell single-cell transcriptomes from the ISB-S CD4 dataset labelled by disease condition.

(b) UMAP visualization of CD4 T cell single-cell transcriptomes labelled by clonal expansion.

(c) Heatmap of differentially expressed markers for all identified clusters (n = 12).

(d) UMAP visualizations highlighting expression levels of individual genes for cell phenotyping.

Figure S6

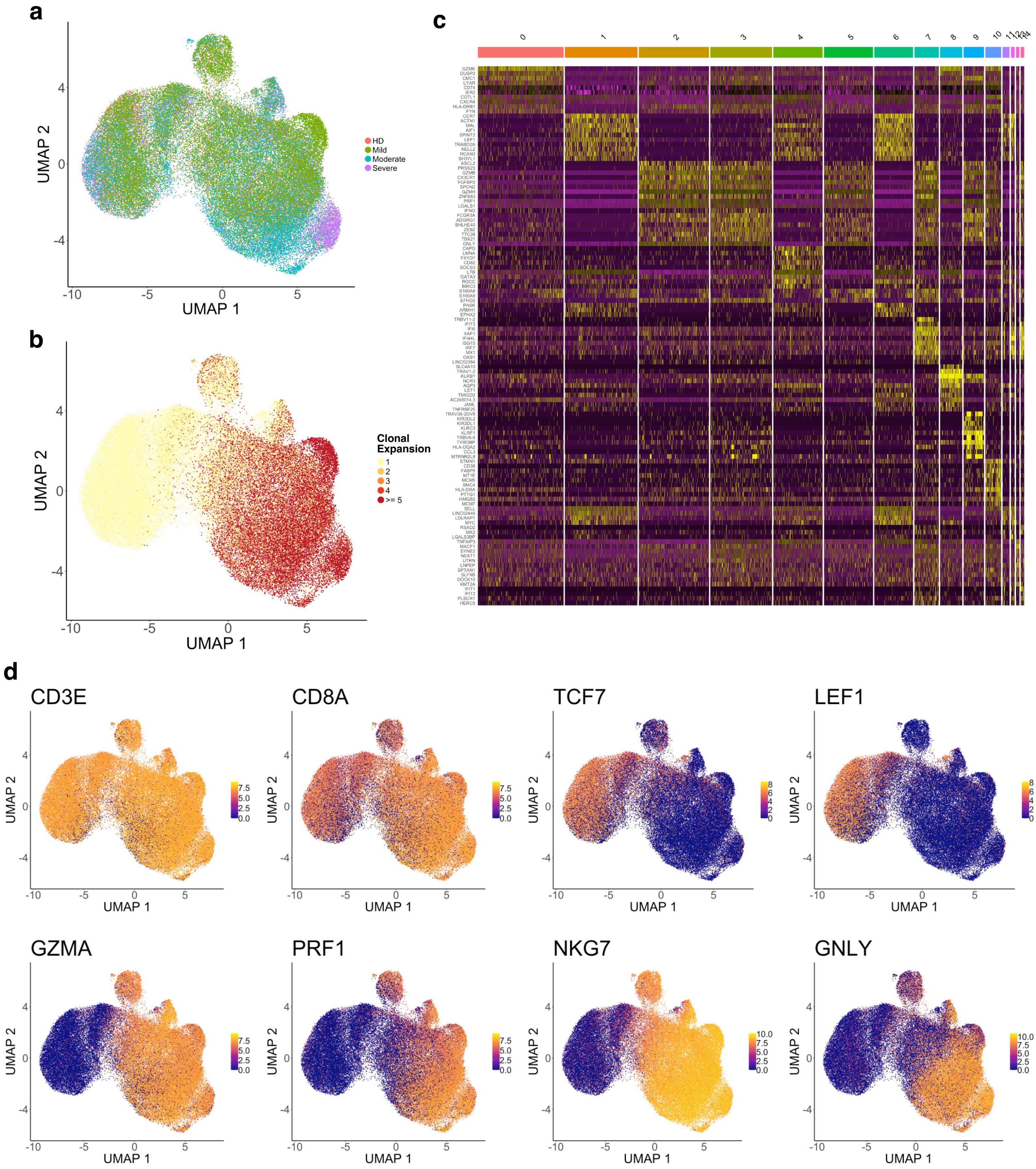
a
UMAP 2
UMAP 1
HD
Mild
Moderate
Severe
b
Clonal Expansion
1
2
3
4
>= 5
c
d
CD3E
CD8A
TCF7
LEF1
GZMA
PRF1
NKG7
GNLY

Figure S6. Additional single-cell transcriptional analyses for CD8 T cells.

(a) UMAP visualization of 70,237 CD8 T cell single-cell transcriptomes from the ISB-S CD8 dataset labelled by disease condition.

(b) UMAP visualization of CD8 T cell single-cell transcriptomes labelled by clonal expansion.

(c) Heatmap of differentially expressed markers for all identified clusters (n = 15).

(d) UMAP visualizations highlighting expression levels of individual genes for cell phenotyping.

Figure S7

a

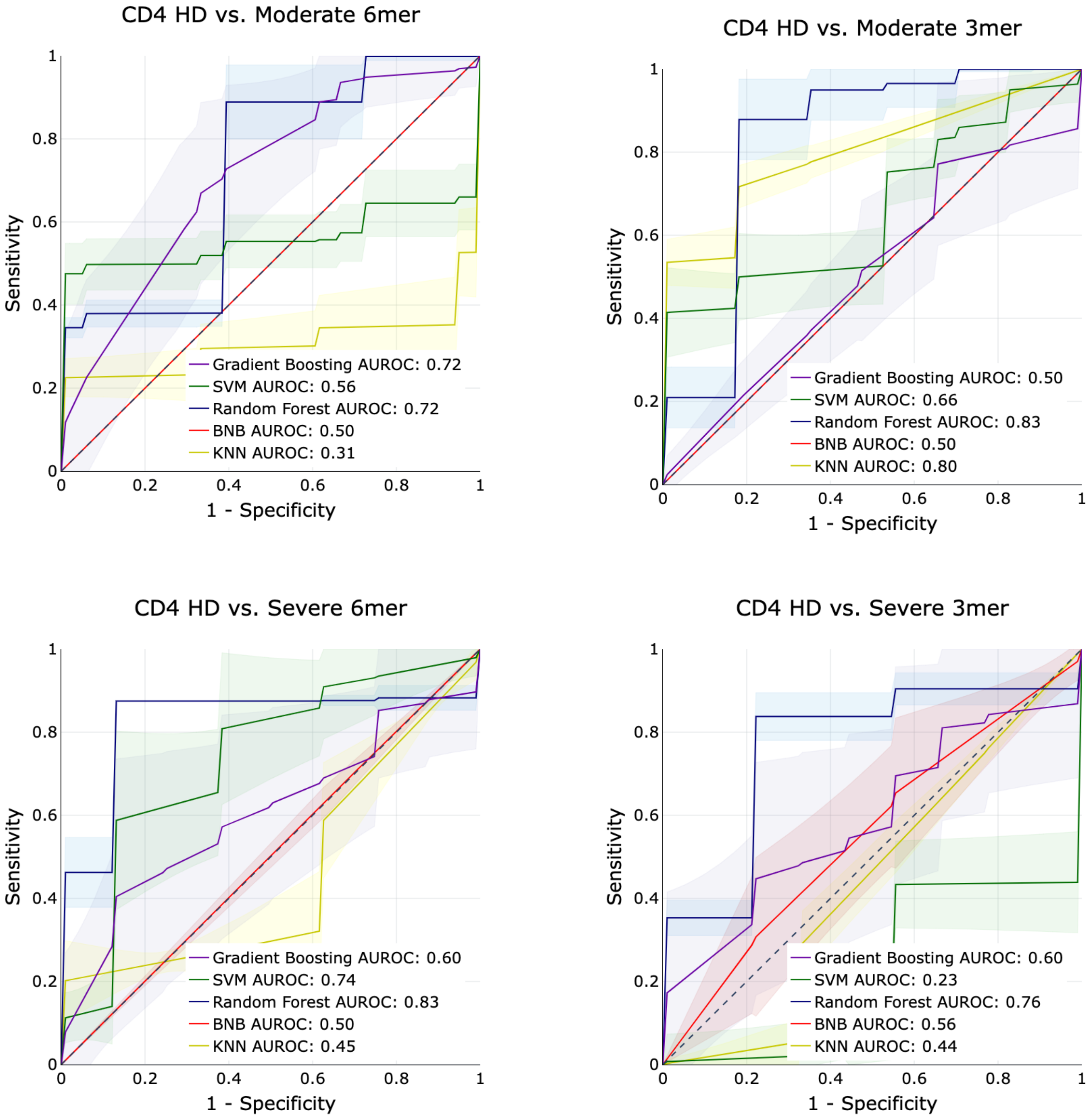
CD4 HD vs. Moderate 6mer
Sensitivity
1 - Specificity
0
0.2
0.4
0.6
0.8
1
Gradient Boosting AUROC: 0.72
SVM AUROC: 0.56
Random Forest AUROC: 0.72
BNB AUROC: 0.50
KNN AUROC: 0.31
CD4 HD vs. Moderate 3mer
Sensitivity
1 - Specificity
Gradient Boosting AUROC: 0.50
SVM AUROC: 0.66
Random Forest AUROC: 0.83
BNB AUROC: 0.50
KNN AUROC: 0.80
CD4 HD vs. Severe 6mer
Sensitivity
1 - Specificity
Gradient Boosting AUROC: 0.60
SVM AUROC: 0.74
Random Forest AUROC: 0.83
BNB AUROC: 0.50
KNN AUROC: 0.45
CD4 HD vs. Severe 3mer
Sensitivity
1 - Specificity
Gradient Boosting AUROC: 0.60
SVM AUROC: 0.23
Random Forest AUROC: 0.76
BNB AUROC: 0.56
KNN AUROC: 0.44

Figure S7. Additional machine learning analyses for CD4 T cell subset.

(a) AUROC curves for five machine learning models using 3-mer and 6-mer representations of TCR repertoire data from the ISB-S CD4 dataset. Models were trained to predict disease severity (moderate, severe) vs healthy donors for CD4 samples. Training and evaluation was performed using 100 repetitions of 5-fold cross-validations per model, average performance +/- 1 standard deviation shown on individual plots.

Supplementary Datasets

Supplementary Dataset

Dataset S1. Metadata for TCR repertoire samples obtained for all datasets used in study.

Dataset S2. Number of clones and unique clonotypes for each sample across datasets.

Dataset S3. CDR3 length statistics for each sample across datasets.

Dataset S4. Diversity statistics including Chao1 estimators, Gini-Simpson indices, and inverse Simpson indices for each sample across datasets.

Dataset S5. V and J gene usage statistics for each sample in Adaptive Biotechnologies datasets.

Dataset S6. V and J gene usage statistics for each sample in ISB-Swedish datasets.

Dataset S7. Principal components analysis results for 3-mer, 4-mer, 5-mer, and 6-mer representations of each sample in ISB-Swedish datasets.

Dataset S8. GLIPH clustering analysis patterns, scores, and statistics for ISB-Swedish datasets by T cell type and disease condition.

Dataset S9. OLGA analysis inputs of structured ISB-Swedish datasets by T cell type and disease condition.

Dataset S10. OLGA analysis output pGen scores of ISB-Swedish datasets by T cell type and disease condition.

Dataset S11. COVID-19-associated clusters in ISB-Swedish datasets by T cell type.

Dataset S12. UMAP coordinates for CD4 and CD8 T cell single-cell transcriptome analyses.

Dataset S13. Cell proportions and counts for clonally expanded groups from CD4 and CD8 T cell single-cell transcriptome analyses.

Dataset S14. Differential gene expression for Cluster 6 vs all other cells in CD4 T cell transcriptome analysis and Expanded group cells vs all other cells in CD8 T cell transcriptome analysis.

Dataset S15. Upregulated and downregulated genes for CD4 and CD8 T cell clonal expansion differential gene expression analysis using threshold q-value < 1e-4.

Dataset S16. DAVID gene ontology biological process annotations for CD4 and CD8 T cell clonal expansion differential gene expression analysis using threshold q-value < 1e-4.

Dataset S17. Average AUROC scores for machine learning models trained to predict disease severity from healthy donors using different k-mer and GLIPH2 representations of TCR repertoires.
