# Supplementary material for: Machine learning identifies T cell receptor repertoire signatures associated with COVID-19 severity
Source: Commun Biol. 2023 Jan 20;6:76. doi: 10.1038/s42003-023-04447-4 (PMC9853487; doi:10.1038/s42003-023-04447-4)
Supplement: Supplementary file 3 — Description of Additional Supplementary Files [file 42003_2023_4447_MOESM3_ESM.pdf]

Description of Additional Supplementary Files

File name: Supplementary Data 1
Description: Metadata for TCR repertoire samples obtained for all datasets used in study.

File name: Supplementary Data 2
Description: Number of clones and unique clonotypes for each sample across datasets.

File name: Supplementary Data 3
Description: CDR3 length statistics for each sample across datasets.

File name: Supplementary Data 4
Description: Diversity statistics including Chao1 estimators, Gini-Simpson indices, and inverse Simpson indices for each sample across datasets.

File name: Supplementary Data 5
Description: V and J gene usage statistics for each sample in Adaptive Biotechnologies datasets.

File name: Supplementary Data 6
Description: V and J gene usage statistics for each sample in ISB-Swedish datasets.

File name: Supplementary Data 7
Description: Principal components analysis results for 3-mer, 4-mer, 5-mer, and 6-mer representations of each sample in ISB-Swedish datasets.

File name: Supplementary Data 8
Description: GLIPH clustering analysis patterns, scores, and statistics for ISB-Swedish datasets by T cell type and disease condition.

File name: Supplementary Data 9
Description: OLGA analysis inputs of structured ISB-Swedish datasets by T cell type and disease condition.

File name: Supplementary Data 10
Description: OLGA analysis output pGen scores of ISB-Swedish datasets by T cell type and disease condition.

File name: Supplementary Data 11
Description: COVID-19-associated clusters in ISB-Swedish datasets by T cell type.

File name: Supplementary Data 12
Description: UMAP coordinates for CD4 and CD8 T cell single-cell transcriptome analyses.

File name: Supplementary Data 13
Description: Cell proportions and counts for clonally expanded groups from CD4 and CD8 T cell single cell transcriptome analyses.

File name: Supplementary Data 14
Description: Differential gene expression for Cluster 6 vs all other cells in CD4 T cell transcriptome analysis and Expanded group cells vs all other cells in CD8 T cell transcriptome analysis.

File name: Supplementary Data 15
Description: Upregulated and downregulated genes for CD4 and CD8 T cell clonal expansion differential gene expression analysis using threshold q-value < 1e-4.

File name: Supplementary Data 16
Description: DAVID gene ontology biological process annotations for CD4 and CD8 T cell clonal expansion differential gene expression analysis using threshold q-value < 1e-4.

File name: Supplementary Data 17
Description: Average AUROC scores for machine learning models trained to predict disease severity from healthy donors using different k-mer and GLIPH2 representations of TCR repertoires.
