# Supplementary material for: Machine learning identifies T cell receptor repertoire signatures associated with COVID-19 severity
Source: Commun Biol. 2023 Jan 20;6:76. doi: 10.1038/s42003-023-04447-4 (PMC9853487; doi:10.1038/s42003-023-04447-4)
Supplement: Supplementary file 5 — Reporting Summary [file 42003_2023_4447_MOESM5_ESM.pdf]

Corresponding author(s): Sidi Chen
Last updated by author(s): Nov 27, 2022

Reporting Summary

Nature Portfolio wishes to improve the reproducibility of the work that we publish. This form provides structure for consistency and transparency in reporting. For further information on Nature Portfolio policies, see our Editorial Policies and the Editorial Policy Checklist.

Statistics

For all statistical analyses, confirm that the following items are present in the figure legend, table legend, main text, or Methods section.

n/a	Confirmed	
☐	☒	The exact sample size (*n*) for each experimental group/condition, given as a discrete number and unit of measurement
☐	☒	A statement on whether measurements were taken from distinct samples or whether the same sample was measured repeatedly
☐	☒	The statistical test(s) used AND whether they are one- or two-sided<br>*Only common tests should be described solely by name; describe more complex techniques in the Methods section.*
☒	☐	A description of all covariates tested
☐	☒	A description of any assumptions or corrections, such as tests of normality and adjustment for multiple comparisons
☐	☒	A full description of the statistical parameters including central tendency (e.g. means) or other basic estimates (e.g. regression coefficient) AND variation (e.g. standard deviation) or associated estimates of uncertainty (e.g. confidence intervals)
☐	☒	For null hypothesis testing, the test statistic (e.g. *F*, *t*, *r*) with confidence intervals, effect sizes, degrees of freedom and *P* value noted<br>*Give P values as exact values whenever suitable.*
☒	☐	For Bayesian analysis, information on the choice of priors and Markov chain Monte Carlo settings
☒	☐	For hierarchical and complex designs, identification of the appropriate level for tests and full reporting of outcomes
☒	☐	Estimates of effect sizes (e.g. Cohen's *d*, Pearson's *r*), indicating how they were calculated

Our web collection on statistics for biologists contains articles on many of the points above.

Software and code

Policy information about availability of computer code

Data collection	No specific software was used to collect data, please refer to manuscript for description of data collection.
Data analysis	R v4.1.0 for custom analyses.<br>Immunarch v0.6.6 for immune repertoire analysis.<br>Seurat v4.0.4 for single cell transcriptome analyses.<br>GLIPH2 irtools v0.01 for TCR specificity clustering.<br>OLGA v1.2.4 for generation probability calculations.<br>Python v3.8.6 and scikit-learn v0.23.1 for machine learning model training.<br>Plotly v5.1.0 for ROC plotting.

For manuscripts utilizing custom algorithms or software that are central to the research but not yet described in published literature, software must be made available to editors and reviewers. We strongly encourage code deposition in a community repository (e.g. GitHub). See the Nature Portfolio guidelines for submitting code & software for further information.

Data

Policy information about availability of data

All manuscripts must include a data availability statement. This statement should provide the following information, where applicable:

- Accession codes, unique identifiers, or web links for publicly available datasets
- A description of any restrictions on data availability
- For clinical datasets or third party data, please ensure that the statement adheres to our policy

Accession codes, unique identifiers, and web links for datasets are described in the manuscript. All data used in this study will be provided to be publicly available with no restrictions.

Field-specific reporting

Please select the one below that is the best fit for your research. If you are not sure, read the appropriate sections before making your selection.

☒ Life sciences ☐ Behavioural & social sciences ☐ Ecological, evolutionary & environmental sciences

For a reference copy of the document with all sections, see nature.com/documents/nr-reporting-summary-flat.pdf

Life sciences study design

All studies must disclose on these points even when the disclosure is negative.

Sample size	Sample sizes were not pre-determined. A total of 4,730,447,888 clones across 2,130 repertoires from multiple studies with COVID-19 and healthy donor TCR sequencing data available at the time this study was initiated were used for analysis.
Data exclusions	Only repertoires that were unable to be processed using Immunarch were excluded from analyses.
Replication	For sequence and motif analyses, statistics and metrics were determined by dataset to ensure reproducibility of findings. For ML training and evaluation, partitioning and cross-validations were repeated 100 times per permutation to ensure reproducibility of model performance.
Randomization	All the samples in the dataset were independent and non-repeating samples. We allocated samples for training the ML models using a stratified k-fold strategy with randomization for training and testing set splitting.
Blinding	Since human patient samples were not used for this study, and group allocations for training were performed automatically (by randomization), blinding was not necessary for this study.

Reporting for specific materials, systems and methods

We require information from authors about some types of materials, experimental systems and methods used in many studies. Here, indicate whether each material, system or method listed is relevant to your study. If you are not sure if a list item applies to your research, read the appropriate section before selecting a response.

Materials & experimental systems

n/a	Involved in the study	
☒	☐	Antibodies
☒	☐	Eukaryotic cell lines
☒	☐	Palaeontology and archaeology
☒	☐	Animals and other organisms
☒	☐	Human research participants
☒	☐	Clinical data
☒	☐	Dual use research of concern

Methods

n/a	Involved in the study	
☒	☐	ChIP-seq
☒	☐	Flow cytometry
☒	☐	MRI-based neuroimaging
